# Investigation of the Anti-Inflammatory Properties of Bioactive Compounds from *Olea europaea*: In Silico Evaluation of Cyclooxygenase Enzyme Inhibition and Pharmacokinetic Profiling

**DOI:** 10.3390/molecules29153502

**Published:** 2024-07-26

**Authors:** Tom C. Karagiannis, Katherine Ververis, Julia J. Liang, Eleni Pitsillou, Evan A. Kagarakis, Debbie T. Z. Yi, Vivian Xu, Andrew Hung, Assam El-Osta

**Affiliations:** 1 Epigenetics in Human Health and Disease Program, Baker Heart and Diabetes Institute, 75 Commercial Road, Prahran, VIC 3004, Australia; 2 Epigenomic Medicine Laboratory at prospED Polytechnic, Carlton, VIC 3053, Australia; 3 Department of Clinical Pathology, The University of Melbourne, Parkville, VIC 3010, Australia; 4 Department of Microbiology and Immunology, The University of Melbourne, Parkville, VIC 3010, Australia; 5 School of Science, STEM College, RMIT University, Melbourne, VIC 3001, Australia; 6 Department of Diabetes, Central Clinical School, Monash University, Melbourne, VIC 3004, Australia; 7 Department of Medicine and Therapeutics, The Chinese University of Hong Kong, Sha Tin, Hong Kong SAR, China; 8 Hong Kong Institute of Diabetes and Obesity, Prince of Wales Hospital, The Chinese University of Hong Kong, 3/F Lui Che Woo Clinical Sciences Building, 30–32 Ngan Shing Street, Sha Tin, Hong Kong SAR, China; 9 Li Ka Shing Institute of Health Sciences, The Chinese University of Hong Kong, Sha Tin, Hong Kong SAR, China; 10 Biomedical Laboratory Science, Department of Technology, Faculty of Health, University College Copenhagen, 2200 Copenhagen, Denmark

**Keywords:** *Olea europaea*, olive phenolics, oleocanthal, oleohydroxypyretol, cyclooxygenase enzymes, anti-inflammatory

## Abstract

In a landmark study, oleocanthal (OLC), a major phenolic in extra virgin olive oil (EVOO), was found to possess anti-inflammatory activity similar to ibuprofen, involving inhibition of cyclooxygenase (COX) enzymes. EVOO is a rich source of bioactive compounds including fatty acids and phenolics; however, the biological activities of only a small subset of compounds associated with *Olea europaea* have been explored. Here, the OliveNet^TM^ library (consisting of over 600 compounds) was utilized to investigate olive-derived compounds as potential modulators of the arachidonic acid pathway. Our first aim was to perform enzymatic assays to evaluate the inhibitory activity of a selection of phenolic compounds and fatty acids against COX isoforms (COX-1 and COX-2) and 15-lipoxygenase (15-LOX). Olive compounds were found to inhibit COX isoforms, with minimal activity against 15-LOX. Subsequent molecular docking indicated that the olive compounds possess strong binding affinities for the active site of COX isoforms, and molecular dynamics (MD) simulations confirmed the stability of binding. Moreover, olive compounds were predicted to have favorable pharmacokinetic properties, including a readiness to cross biological membranes as highlighted by steered MD simulations and umbrella sampling. Importantly, olive compounds including OLC were identified as non-inhibitors of the human ether-à-go-go-related gene (hERG) channel based on patch clamp assays. Overall, this study extends our understanding of the bioactivity of *Olea-europaea*-derived compounds, many of which are now known to be, at least in part, accountable for the beneficial health effects of the Mediterranean diet.

## 1. Introduction

**Figure 1:**
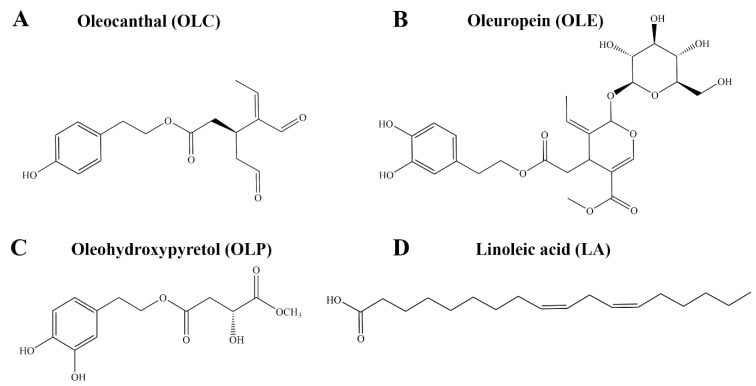
Chemical structures of bioactive compounds from *Olea europaea*. This includes the phenolic compounds (**A**) OLC, (**B**) OLE, and (**C**) OLP, as well as the fatty acid (**D**) LA. OLC is a known inhibitor of the COX-1 and COX-2 enzymes.

Oleocanthal (OLC) is a phenolic compound that contributes to the pungency of extra virgin olive oil (EVOO) [1,2]. The concentration of OLC in EVOO is variable, ranging from as little as 0.2 mg/kg to 498 mg/kg [3,4]. OLC has been reported to share similar throat-irritant properties as the non-steroidal anti-inflammatory drug (NSAID) ibuprofen (Figure 1A) [1,2]. In a landmark study by Beauchamp et al., the anti-inflammatory properties of OLC were explored [1]. OLC was found to share similar pharmacological effects as ibuprofen, with the olive-derived compound dose-dependently inhibiting the activity of cyclooxygenase-1 (COX-1) and cyclooxygenase-2 (COX-2) in vitro [1]. The findings from the study provided insight into the possible health benefits of EVOO, which is a major component of the Mediterranean diet [1,5]. Since the study by Beauchamp et al., the anti-inflammatory properties of bioactive phenolic compounds from olive oil including oleuropein (OLE) and hydroxytyrosol (HT) have been investigated (Figure 1B) [6,7,8].

The arachidonic acid pathway plays an important role in regulating the inflammatory response [9]. Polyunsaturated fatty acids obtained from dietary sources are metabolized into the eicosanoid precursors arachidonic acid (AA) and eicosapentaenoic acid (EPA) [10]. The eicosanoid precursors are released from cell membranes by phospholipases [11]. The ω-6 polyunsaturated fatty acid, arachidonic acid, can be metabolized through the action of cyclooxygenases (COXs), lipoxygenases (LOXs), and cytochrome P450 (CYP) enzymes [11,12].

The COX-1 isoform is constitutively expressed in most tissues, while COX-2 is predominantly induced by pro-inflammatory activities [13]. The initial COX reaction involves the oxygenation of arachidonic acid to form prostaglandin endoperoxide G2 (PGG_2_) [11]. This is followed by a peroxidase (POX) reaction that reduces PGG_2_ to prostaglandin endoperoxide H_2_ (PGH_2_) [11]. PGH_2_ serves as the precursor for the synthesis of prostanoids including prostaglandins, prostacyclins, and thromboxanes [10,11]. The COX enzymes function as homodimers, with each monomer consisting of three structural domains: an N-terminal epidermal growth factor (EGF)-like domain, a membrane-binding domain, and a C-terminal globular catalytic domain [12,14]. The catalytic domain comprises the COX and POX active sites [12,14,15]. The COX active site accommodates substrates or inhibitors, while the POX active site contains a heme cofactor [12,14,15].

Furthermore, LOX enzymes catalyze the formation of leukotrienes [11,16]. The LOX enzymes oxygenate arachidonic acid to generate hydroperoxy derivatives and exhibit lipohydroperoxidase activity [16]. Various isoforms of LOX have been identified, including human reticulocyte 15-lipoxygenase (15-LOX) [16]. Highly expressed in airway epithelial cells, 15-LOX plays a role in promoting immune cell migration and has been implicated in airway inflammatory diseases [17]. In contrast to the COX-1 and COX-2 enzymes, ibuprofen and OLC have been reported to have no effect on the activity of 15-LOX in vitro [1].

NSAIDs, which target the COX enzymes, are widely used to treat pain and reduce inflammation [18]. The health benefits associated with the use of NSAIDs, such as ibuprofen and aspirin, at lower doses have been reported [1,19]. Conversely, studies have shown that the long-term use of NSAIDs can result in adverse effects including gastrointestinal complications [20]. Due to the gastrointestinal side effects associated with classical non-selective NSAIDs, a number of COX-2-selective inhibitors were developed [21,22,23]. Subsequent studies revealed that the selective COX-2 inhibitors, rofecoxib and valdecoxib, were associated with increased cardiovascular risk, leading to the withdrawal of the drugs from the market [22,23].

As a result, there is an interest in identifying and developing alternative COX inhibitors with enhanced therapeutic efficacy and minimal side effects [23,24,25]. The inhibitory activity of dietary polyphenols and their derivatives against COX-1 and COX-2 have been reported, highlighting the potential for natural compounds to be explored as novel COX inhibitors [26]. Despite the identification and characterization of more than 200 phenolic compounds from *Olea europaea*, approximately half are commercially available and only a relatively small fraction have been evaluated in models of human disease.

The OliveNet^TM^ database is a curated library of over 600 compounds from *Olea europaea*, including 222 phenolic compounds [27]. Here, using in vitro enzymatic assays, we aimed to evaluate the inhibitory activity of a selection of phenolic compounds and fatty acids from the OliveNet^TM^ database against the COX-1, COX-2, and 15-LOX enzymes. The mechanisms of action of the well-known COX inhibitor OLC and the novel phenolic compound oleohydroxypyretol (OLP) (Figure 1C) were investigated further using in silico methods. Molecular docking and molecular dynamics (MD) simulations were performed to examine the binding characteristics of OLC and OLP against the COX and 15-LOX enzymes. Furthermore, the pharmacokinetic properties of OLC and OLP were assessed using computational tools and in vitro patch clamp assays.

## 2. Results and Discussion

### 2.1. In Vitro Inhibitory Activity of Olea europaea Compounds against COX and LOX Enzymes

Direct enzymatic assays were performed to evaluate the inhibitory activity of a selection of phenolic compounds from the OliveNet^TM^ database against the COX-1, COX-2, and 15-LOX enzymes. In addition to OLC, which was previously identified as an inhibitor of the COX enzymes, the well-known phenolic compounds HT, hydroxytyrosol acetate (HTA), tyrosol (TYR), OLE, and homovanillic acid (HVA) were evaluated [1]. The novel phenolic compound OLP was also examined [28,29]. Due to the involvement of the COX and LOX enzymes in the metabolism of arachidonic acid, the potential inhibitory activity of a selection of fatty acids from the OliveNet^TM^ database was investigated. Oleic acid (OA) is the main monounsaturated fatty acid found in olive oil (55–83%), with linoleic acid (LA) and palmitic acid (PA) being detected in smaller quantities (Figure 1D) [30,31].

**Figure 2:**
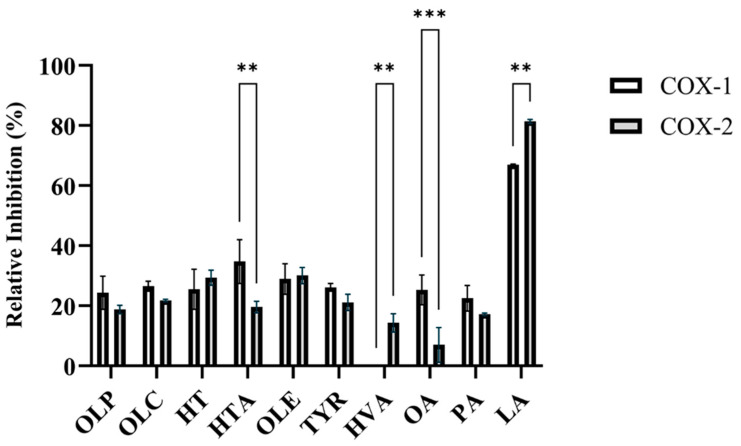
Relative inhibition (%) of the COX-1 and COX-2 enzymes by olive-derived compounds. The inhibitory activity of the phenolic compounds OLP, OLC, HT, HTA, OLE, TYR, and HVA, as well as the fatty acids OA, PA, and LA, against COX-1 and COX-2 can be seen. The data presented denotes the mean ± SD from duplicate assays. ** *p* ≤ 0.01 and *** *p* ≤ 0.001 quantified using a 2-way ANOVA with Šídák’s multiple comparison test.

SC560 and celecoxib were used as positive control inhibitors for COX-1 and COX-2, respectively. At a concentration of 1 nM, SC560 inhibited 75 ± 3.1% of COX-1 activity (Table S1). Celecoxib inhibited 91 ± 0.4% of COX-2 activity at a concentration of 1 µM (Table S1). In accordance with the study by Beauchamp et al., OLC was found to inhibit the activity of the COX isoforms (Figure 2, Table S1). As seen in Figure 2, OLC inhibited 26.6 ± 1.7% and 21.8 ± 0.4% of COX-1 and COX-2 activity, respectively. Like OLC, the phenolic compounds HT, HTA, OLE, OLP, and TYR inhibited the enzymatic activity of COX-1 and COX-2 (Figure 2, Table S1).

OLE inhibited 28.9 ± 5.1% of COX-1 and 30.1 ± 2.7% of COX-2 activity (Figure 2, Table S1). OLE has previously been shown to have inhibitory activity against the COX enzymes and decreased the expression of COX-2 in lipopolysaccharide-treated colonic mucosa from ulcerative colitis patients [6,32]. At 12.5 µM, the relative inhibitory activity of the novel phenolic compound OLP was measured to be 24.4 ± 5.5% for COX-1 and 18.8 ± 1.4% for COX-2 (Figure 2, Table S1). HTA (34.8 ± 7.3%) and OA (25.3 ± 5.0%) exhibited greater inhibitory activity against COX-1 in comparison to COX-2 (Figure 2, Table S1). HVA was found to inhibit COX-2 (14.4 ± 3.0%), while having no inhibitory activity against COX-1 (Figure 2, Table S1). Furthermore, the direct enzymatic assays revealed the potent inhibitory activity of LA against the COX-1 and COX-2 isoforms (Figure 2, Table S1). Several studies have assessed the COX-inhibitory effects of fatty acids including LA, OA, and PAs [33,34,35,36].

The inhibitory activity of the phenolic compounds and fatty acids from the OliveNet^TM^ database against 15-LOX was also investigated (Table S1). Nordihydroguaiaretic acid (NDGA) is a phenolic compound that has antioxidant activity and is an established inhibitor of 15-LOX [37,38]. NDGA was found to inhibit 100% of 15-LOX activity at 100 µM (Table S1). At a concentration of 12.5 µM, the phenolic compounds and fatty acids exhibited minimal inhibitory activity against 15-LOX (Table S1). The results are consistent with the findings from the study by Beauchamp et al., as OLC was reported to have no effect on the activity of 15-LOX in vitro [1].

### 2.2. In Silico Screening of Olive-Derived Compounds against the Active Site of COX and LOX Enzymes

Using AutoDock Vina, molecular docking was subsequently performed to examine the binding characteristics of OLC and the novel phenolic compound OLP against the active sites of the COX-1 and COX-2 dimers (Table 1, Figures S1 and S2) [39]. OLC was predicted to bind to the chain A and chain B subunits of COX-1 with an affinity of −7.8 and −7.7 kcal/mol, respectively. OLC formed a π–π stacking interaction with the hydrophobic residue F209 in both subunits of COX-1, as well as a hydrogen bond with the hydrophobic residue Y385 of chain B (Table 1). OLP was predicted to bind with an affinity of −7.2 and −7.5 kcal/mol to the chain A and chain B subunits of COX-1, respectively. The positively charged residue R120 was predicted to form hydrogen bonds with OLP in each subunit (Table 1). An additional hydrogen bond was detected between OLP and Y355 of chain B (Table 1).

OLC was predicted to bind to the chain A and chain B subunits of COX-2 with an affinity of −5.8 and −7.2 kcal/mol, respectively. The binding affinity of OLP for chain A and chain B was −6.9 and −7.5 kcal/mol, respectively. OLC and OLP were predicted to form hydrogen bonds with the positively charged residue R120 (Table 1). OLC was also predicted to form hydrogen bonds with Y385 and Y355, while OLP formed hydrogen bonds with Y355 and G526 (Table 1).

When examining the chemical structures of OLC and OLP, it was evident that the hydroxyl and carbonyl groups were predominantly forming hydrogen bonds with the active site residues of COX-1 and COX-2. Based on a previous structure–activity relationship study by Ribeiro et al., the phenolic hydroxyl groups of cinnamic acid derivatives were predicted to be essential for the inhibitory activity against the COX-1 and COX-2 isoforms [40]. Furthermore, Honmore et al. investigated the anti-inflammatory and antioxidant activity of isolated compounds from *Alpinia officinarum* rhizomes [41]. To explore the mechanisms of action of galangin and 5-hydroxy-7-(4″-hydroxy-3″-methoxyphenyl)-1-phenyl-3-heptanone, the compounds were screened against the active site of COX-2 [41]. Hydrogen bonding and π–π stacking interactions with key residues including Y355, Y385, and S530 were reported to function as anchors, guiding the inhibitors into the active site [41].

Similar to the natural substrate arachidonic acid, the majority of classical NSAIDs contain a carboxylic acid group that interacts with the conserved residue R120 at the entrance of the active site channel [42]. In COX-1, the interaction between the carboxylate end of the substrate and R120 orientates the aromatic portion of NSAIDs toward Y385, which is a key catalytic residue involved in the initial step of the oxygenation reaction of arachidonic acid [42]. In comparison to COX-1, COX-2 has broader substrate specificity and substrate binding is less dependent on the interaction with R120 [12,43].

Due to the potent in vitro inhibitory activity of LA, the fatty acid was also selected for further analysis. A variety of n-3 and n-6 18–22 carbon fatty acids can compete with arachidonic acid, which is the preferred substrate for COX-1 and COX-2, for binding to the active site [44]. As a result, the formation of 2-series prostaglandins derived from arachidonic acid is inhibited [44]. LA was predicted to bind to the chain A and chain B subunits of COX-1 with an affinity of −7.3 and −7.2 kcal/mol, respectively. The binding affinity of LA for the chain A and chain B subunits of COX-2 was −6.2 and −6.9 kcal/mol, respectively. A hydrogen bond was detected between LA and V349 of the COX-1 chain A subunit, as well as R120 of the COX-2 subunits (Table 1). For both COX-1 and COX-2, the carboxylate group of LA was positioned in proximity to the positively charged residue R120.

Previous structural studies have shown that alternative fatty acid substrates bind to the COX-1 and COX-2 active sites in an L-shaped configuration, similar to that observed for arachidonic acid [44]. The carboxylate group is positioned near the side chain of R120 and Y355 at the opening of the channel, while the ω-end of the fatty acid binds in a hydrophobic groove [44]. In addition to the catalytically productive conformation, arachidonic acid and eicosapentaenoic acid have also been found to adopt a nonproductive pose in a subunit of COX-2 [12]. The fatty acids are inverted within the channel, with the ω end directed toward the opening and the carboxylate end stabilized by interactions with Y385 and S530 [12].

Molecular docking was also employed to investigate the binding characteristics of OLC, OLP, and LA against the active site of the 15-LOX isoform (Figure S3). Mammalian LOXs share similar structural features, as they are composed of an N-terminal β-barrel domain and larger C-terminal α-helical catalytic domain that contains the non-heme iron [45,46,47]. In comparison to COX-1 and COX-2, the olive-derived compounds were predicted to bind with a weaker affinity to the active site of 15-LOX: OLC (−6.3 kcal/mol), OLP (−6.3 kcal/mol), and LA (−6.1 kcal/mol). OLC and OLP were predicted to form hydrogen bonds with R402 and Q595 of 15-LOX. Moreover, OLP was predicted to form a hydrogen bond with I662.

### 2.3. Protein Dynamics of COX Enzymes in Response to Olive-Derived Compounds

**Figure 3:**
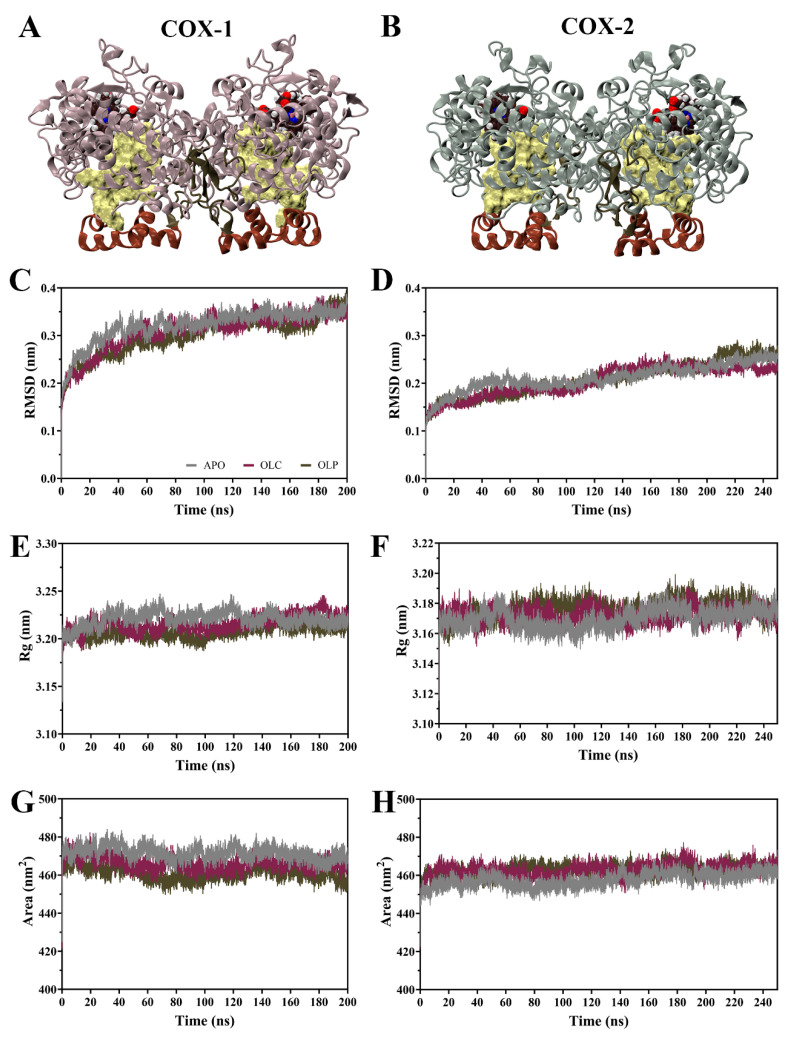
Molecular dynamics (MD) simulations of COX-1 and COX-2 homodimers bound with olive-derived compounds. (**A**) Structure of COX-1 and (**B**) COX-2 homodimers. Each monomer consists of an epidermal growth factor domain (green), a membrane-binding domain (brown), and a catalytic domain. The heme cofactor is shown in van der Waals representation, and the binding pocket is shown in surface representation in yellow. (**C**) Root mean square deviation (RMSD) of the protein backbone of COX-1 and (**D**) COX-2 with respect to its initial structure. (**E**) Radius of gyration (Rg) of the protein backbone for COX-1 and (**F**) COX-2. (**G**) Solvent-accessible surface area of the protein surface for COX-1 and (**H**) COX-2. Data is shown as an average of three runs, with the ligand-free protein (APO) shown in gray, and the enzymes bound with OLC in purple and OLP in green.

The dynamic behavior of COX enzymes binding to olive compounds were studied using MD simulations performed with GROMACS [48,49,50]. Simulations were performed on the dimeric COX-1 and COX-2 enzymes with an olive compound, either OLC or OLP, bound to each monomer, as well as the ligand-free enzyme (APO) (Figure 3A,B). Simulations for COX-1 were performed for 200 ns, the same being 250 ns for COX-2 due to it taking a longer time to equilibrate (Figure 3).

Root mean square deviation (RMSD) of the protein backbone indicates that the COX-1 systems reached equilibrium after 100 ns (Figure 3C,D). For COX-2, systems did not equilibrate until 150 ns, hence the simulations were extended for an additional 50 ns. Subsequent calculations were performed for all systems on 100 ns of equilibrated production runs for analysis. Following equilibration, average RMSD values were similar within COX-1 (APO = 0.34, OLC = 0.34, OLP = 0.33 nm) and COX-2 systems (APO = 0.24, OLC = 0.23, OLP = 0.25 nm).

Radius of gyration (Rg) was similar across systems (Figure 3E,F), with an average Rg of 3.22 nm for COX-1 and 3.17 nm for COX-2 systems, indicating minor differences between COX-1 and COX-2 but similarities between APO and ligand-bound systems. This suggests that binding of olive compounds does not affect overall compactness of the dimeric complexes. Similarities were also observed in solvent-accessible surface area (SASA) between all the systems (COX-1 APO = 471, OLC = 466, OLP = 461 nm^2^; COX-2 APO = 460, OLC = 465, OLP = 464 nm^2^) (Figure 3G,H), suggesting an absence of large structural changes in the protein complexes in response to ligand binding.

**Figure 4:**
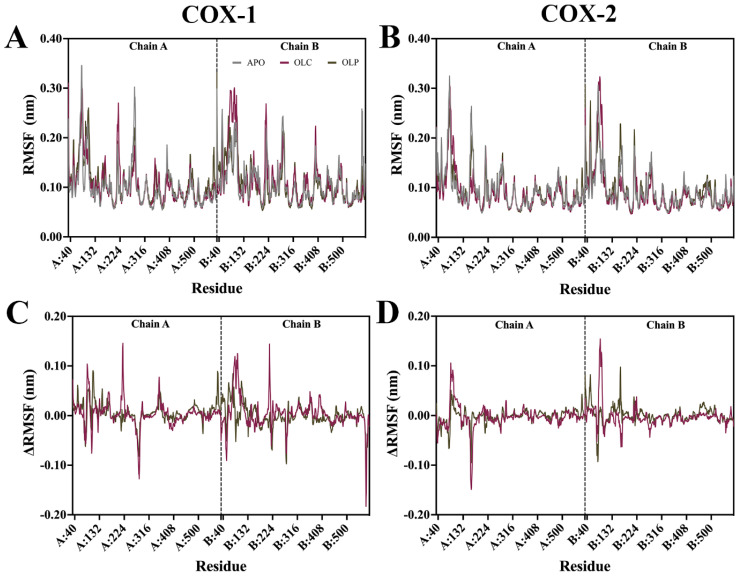
Root mean square fluctuation (RMSF) of the protein backbone for COX-1 and COX-2 bound with olive-derived compounds. (**A**) RMSF for COX-1 backbone and (**B**) COX-2 is shown as an average of three runs following equilibration of the trajectory. (**C**) Difference in RMSF of protein backbone with APO values are subtracted from OLC- and OLP-bound COX-1 and (**D**) COX-2. The vertical dashed line indicates the residues that form part of chain A and chain B in the dimeric structure.

Root mean square fluctuation (RMSF) analysis was performed on the protein backbone, examining the flexibility of residues throughout the simulation (Figure 4). The difference in RMSF (ΔRMSF) was calculated by subtracting APO values from ligand-bound values to examine differences that may be attributed to binding of olive compounds. In general, the N-terminal EGF-like domain and membrane-binding domains (MBDs) of each monomer demonstrated a higher RMSF than the rest of the protein, while the catalytic domain had a lower overall RMSF. Catalytic residues in COX-1 and COX-2 did not display large fluctuations. Peaks in RMSF occurred largely in peripheral loops, such as at regions spanning residues 268 to 283 in chain A of COX-1, reaching 0.30 nm, and 0.25 nm at 156 to 168 of COX-2 chain B. A loop spanning residues 212 to 225 located near the peroxidase site in both chains of COX-1 and COX-2 demonstrated a high RMSF of around 0.18 nm.

Changes in ΔRMSF were minor across all systems. Changes in ΔRMSF outside the EGF and MBD domains were more pronounced in COX-1 than in COX-2. At residues 350 to 358 of COX-1 chain A, there was a ΔRMSF of 0.08 nm for OLC, which is located within the binding site, suggesting minor shifts in the protein to accommodate ligand binding.

### 2.4. Dynamic Interactions of Olive-Derived Compounds within the Catalytic Site of COX Enzymes

**Figure 5:**
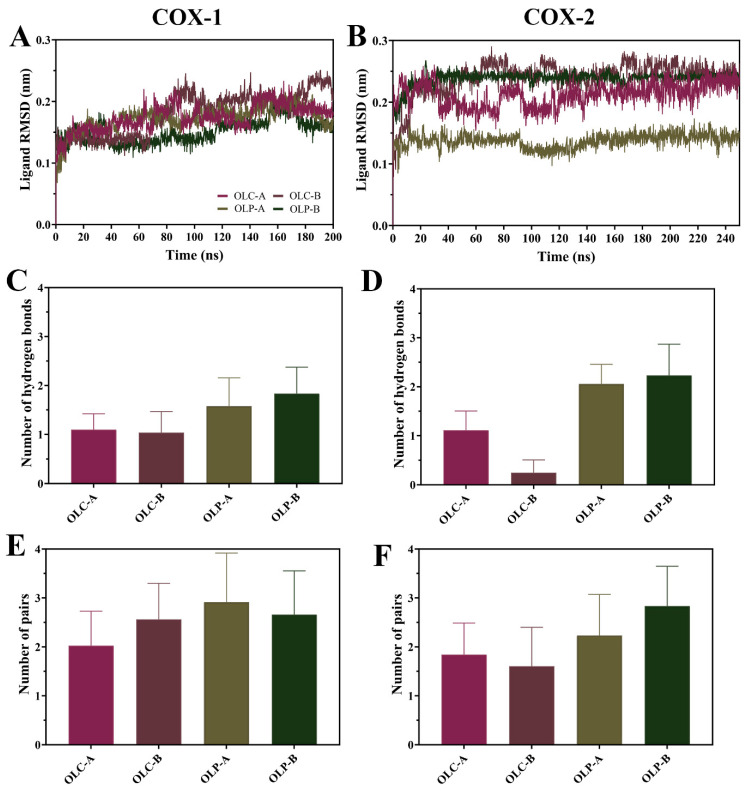
Dynamics of olive-derived compounds bound to the active site of COX-1 and COX-2. (**A**) RMSD of OLC and OLP bound to the catalytic site of each chain of COX-1 and (**B**) COX-2 with respect to its initial structure. (**C**) Number of hydrogen bonds between olive compounds and COX-1 and (**D**) COX-2. (**E**) Number of pairs within 0.35 nm between olive compounds and COX-1 and (**F**) COX-2. Data is shown as mean ± SD.

To assess the stability of olive-derived ligands within the active site of the COX enzymes, ligand RMSD was calculated (Figure 5A,B). Following equilibration, average values were typically under 0.20 nm for OLC and OLP bound to COX-1. For COX-2, average values were under 0.25 nm, with the exception of OLP bound to chain A having an average RMSD of 0.14 nm. This indicates that the ligands were stable with respect to their initial position within the catalytic site throughout the simulations.

There was a higher average number of hydrogen bonds formed with OLP compared to OLC for both COX-1 and COX-2 throughout the simulations (Figure 5C,D). Interestingly, the number of protein–ligand atom pairs within 0.35 nm is higher and more similar across the ligands. This suggests that the binding of OLC is not necessarily driven by the formation of hydrogen bonds, unlike OLP. This is consistent with the OLP having multiple additional hydroxyl groups compared to OLC, with OLP having six hydrogen donors and seven hydrogen acceptors, compared to OLC with two donors and five acceptors.

The binding free energy between olive-derived compounds and the COX enzymes was calculated using MM-PBSA (Table 2). This showed that OLC had a stronger binding affinity than OLP for both COX-1 and COX-2, which is in agreement with the inhibitory activity presented in Figure 2. For COX-1, the calculated ΔG_bind_ for OLP was −23.57 ± 1.99 kcal/mol in chain A and −21.83 ± 0.90 kcal/mol in chain B, compared to OLC where the ΔG_bind_ was −17.55 ± 0.56 kcal/mol in chain A and −15.83 ± 1.75 kcal/mol for chain B. These values were similar in COX-2; however, OLC bound to chain B of COX-2 had a relatively lower ΔG_bind_ of −13.45 ± 1.45 kcal/mol. The difference in binding energy between monomers lends support to growing evidence that COX enzymes may act as functional heterodimers, where one monomer acts as a catalytic subunit while the second monomer serves a regulatory role [51]. The predominant driving force for binding of OLC and OLP to COX-1 and COX-2 enzymes were van der Waals energy contributions (Table 2), due to the hydrophobic residues lining the active site. OLP has stronger energy contributions from electrostatic forces compared to OLC, indicative of the greater number of hydrogen bonds shown in Figure 5C,D.

**Figure 6:**
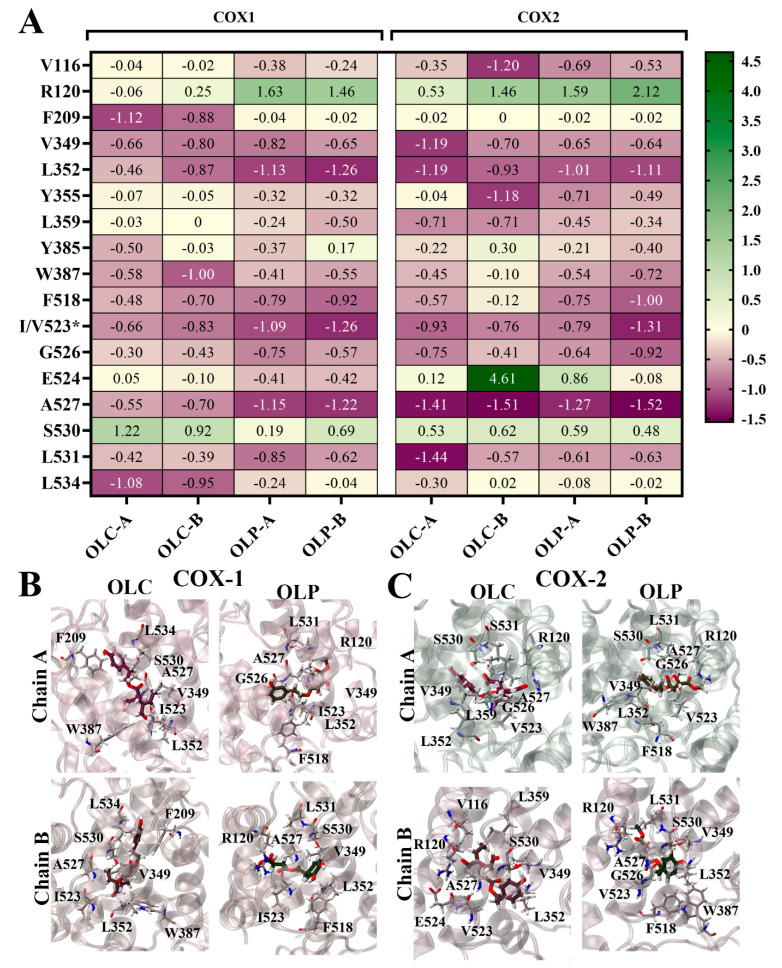
Per-residue contributions to binding energy of olive-derived compounds to COX-1 and COX-2. (**A**) Heatmap of key residues contributing to binding energy for OLC and OLP bound to each chain of COX-1 and COX-2 homodimers. Energy contributions are shown in kcal/mol as an average of three independent binding free energy calculations using MM-PBSA. The asterisk* indicates amino acid difference between COX isoforms. (**B**) OLC and OLP binding to COX-1 and (**C**) COX-2, with key residues highlighted in stick representation. Oxygens atoms are red, nitrogen atoms are blue, and hydrogen atoms are white.

To identify the residues contributing to ligand binding, the binding energy was decomposed on a per-residue basis (Figure 6). Energy contributions from key residues are depicted as a heatmap in Figure 6A, and the ligands bound to the active site of the COX isoenzymes are visualized in Figure 6B,C. The major residues contributing favorably to binding included L352 and residue 523 (isoleucine in COX-1 and valine in COX-2). Some residues with charged and polar side chains contributed unfavorably to ligand binding, including active site residues R120 and S530. E524 strongly contributed unfavorably to OLC binding to chain B of COX-2, accounting for its overall weaker ΔG_bind_. The stronger ΔG_bind_ of OLC binding to COX-1 can be attributed to strong energy contributions from hydrophobic residues F209 and L534 (Figure 6A), as well as F215, V344, and Y348 (Figure S4). Binding of OLC to COX-2 was assisted by favorable energy contributions from L359 and L532. For OLP, F518 made favorable energy contributions. This residue is located in the side pocket of COX-2, which is exploited by COX-2-selective inhibitors [52].

### 2.5. Bioactivity and Toxicity of OLC and OLP

In silico pharmacokinetic prediction tools were utilized to evaluate the predicted absorption, distribution, metabolism, excretion, and toxicity (ADMET) properties of OLC and the novel phenolic compound OLP. Based on the results from the QikProp analysis, OLC and OLP were predicted to be permeable across Caco-2 cells and orally bioavailable (Table S2) [53]. Similarly, SwissADME predicted OLC and OLP to be absorbed by the gastrointestinal tract; however, the phenolic compounds were unable to cross the blood–brain barrier (BBB) (Table S2) [54]. Moreover, OLC and OLP were not predicted to be substrates of P-glycoprotein (P-gp) and were non-inhibitors of the CYP450 isoenzymes (Table S2).

Regarding in vivo experiments and potential further progression to clinical trials, inhibition of human ether-à-go-go-related gene (hERG) K^+^ channels represents a major obstacle given the risks associated with QT prolongation and propensity for severe cardiac events [55]. The QPloghERG values, which are a measure of the IC_50_ for blockage of hERG K^+^ channels, for OLC and OLP were predicted to be −5.2 and −4.7, respectively (concern < −5). To validate the results from the ADMET analysis, hERG patch clamp assays were performed (Figure S5). Similar results were obtained from two independent experiments, which showed that OLC and OLP were not hERG inhibitors. At the highest concentration, OLC (10 μM) and OLP (100 μM) inhibited 44% and 10% channel activity, respectively. Verapamil, which is a known hERG inhibitor, was found to inhibit 100% channel activity at 10 μM (Figure S5).

### 2.6. Membrane Permeability of Compounds Derived from Olea europaea

**Figure 7:**
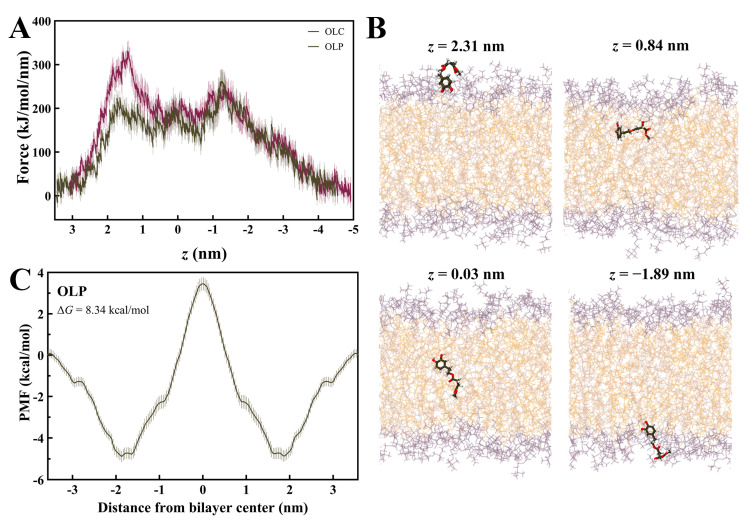
Membrane permeability of olive-derived compounds. (**A**) Mean force profile for OLC and OLP passing through a DOPC membrane along the membrane normal (*z*). Data is shown as the mean ± SD of ten independent pulling simulations. (**B**) Permeation of OLP through the DOPC bilayer. (**C**) Symmetrized potential of mean force (PMF) profile for OLP passing through DOPC membrane. Oxygens atoms are red and hydrogen atoms are white.

Steered MD simulations were performed using GROMACS to calculate the force required for OLC and OLP to penetrate a DOPC membrane bilayer, as previously performed with other olive-derived compounds [48,49,50,56]. The profile for the average pulling force over ten independent SMD simulations for OLC and OLP is shown in Figure 7A. It can be observed that energy barriers are encountered as lipids enter the membrane, with a higher initial force required for OLC, a plateau in energy as the ligands pass through the center of the bilayer, and gradual decrease in force as the ligands exit the membrane. OLP demonstrates maximum forces of 224 k/mol/min and 262 kJ/mol/min as it enters and exits the bilayer, respectively, while plateauing at 180 kJ/mol/min passing through the bilayer. As observed in Figure 7B, the phenol head group of OLP is orientated downwards as it approaches the membrane before assuming a flatter conformation as it enters the bilayer. As it passes through the membrane, the tail is gradually pointing downwards to exit tail-first.

The PMF curve for OLP was calculated using umbrella sampling (Figure 7C). The PMF was determined by pulling the ligand across the entire membrane and then symmetrizing the resulting profile [57]. Initial unsymmetrized profiles had simulation times incrementally increased by 10 ns for a maximum of 100 ns for each window (Figure S6). To determine equilibration time, 10 ns increments were successively discarded from each window until the force profiles converged. Convergence was achieved after 50 ns of equilibration, hence the final profile had 50 ns of sampling for each window. The resulting PMF curve for the penetration of OLP through the DOPC bilayer yielded a ΔG of 8.34 kcal/mol. This value is in accordance with Boggara et al., who found the ΔG of ibuprofen to be 8.75 ± 0.72 kcal/mol under similar conditions [58].

## 3. Materials and Methods

### 3.1. Preparation of Protein Structures

Homology modeling was used to construct the initial structure of COX-1, as previously described [24]. The heme group was retained in the structure and template crystallographic transformations were applied to the homology model to generate a dimeric complex of COX-1. The crystal structure of COX-2 was obtained from the RCSB Protein Data Bank (PDB ID: 5F1A) [24,59]. The cobalt atom present in the crystallographic porphyrin group was replaced with iron [60]. The dimeric COX complexes underwent energy minimization using GROMACS 2018.4 [48,49] with the steepest-descent gradient method, and an energy convergence level of 100 kJ/mol/nm.

The structure of 15-LOX was constructed using homology modeling. The amino acid sequence of human 15-LOX was obtained from UniProt and a similarity search was performed using the protein–protein basic local alignment search tool (blastp) [61,62]. Based on the sequence identity (81.1%), the structure of the rabbit reticulocyte 15S-lipoxygenase (PDB ID: 2P0M) was used as the template [46]. Homology modeling was performed using Modeller 9.20 [63]. The stereochemical quality of the selected model was evaluated using PROCHECK (Figure S7) [64].

### 3.2. Molecular Docking Procedure

The phenolic compounds and fatty acids utilized in this study were predominantly sourced from the OliveNet^TM^ library [27]. The chemical structures of OLC and LA were obtained from the National Center for Biotechnology Information (NCBI) PubChem database, while the chemical structure of OLP was drawn using Chem3D 21.0.0 (PerkinElmer, Waltham, MA, USA) [65]. The protein structures of COX-1, COX-2, and 15-LOX were imported into AutoDockTools-1.5.7 and were prepared as macromolecules [66]. The chemical structures of the phenolic compounds were imported into PyRx and were energy minimized using the universal force field through Open Babel (v. 2.2.3) [67,68].

AutoDock Vina was used to perform molecular docking at an exhaustiveness of 2048 [39]. For both COX-1 and COX-2, the receptor grid was centered on the active site residues Y385, S530, R120, and Y355 on each chain [24]. Residues F352, R402, F414, and I417 were used to generate the receptor grid for the open conformation of 15-LOX [69]. The receptor grids were 20 × 20 × 20 Å in size. The binding affinities (kcal/mol) and non-covalent protein–ligand interactions were subsequently analyzed using Visual Molecular Dynamics 1.9.3 and Maestro 13.2 [70,71].

### 3.3. Molecular Dynamics Simulations (MD) Simulations of COX Enzymes

MD simulations were performed for OLC and OLP bound to each chain of the dimeric COX-1 and COX-2 complexes. Docked ligands served as starting structures for simulation, as described above. Simulations were performed as previously described [24] with GROMACS 2018.2 [48,49] and the CHARMM36 force field [72], with ligand topologies generated using CHARMM General Force Field (CGenFF) [73]. The protein–ligand complexes were solvated in a dodecahedral box with the TIP3P water model [74] with a minimum distance of 1.0 nm between protein atoms to the closest box edge. Systems were neutralized and salted to 0.15 M NaCl and underwent steepest-descent gradient energy minimization. Equilibration was performed using canonical (NVT) and isothermal–isobaric (NPT) ensembles for 100 ps, maintaining a temperature of 310 K with a modified Berendsen thermostat [75] and a pressure of 1.0 bar with the Parrinello–Rahman barostat [76]. The LINCS algorithm [77] was used to constrain bond lengths, and calculation of long-range electrostatics was performed with the particle-mesh Ewald scheme (PME) [78] with grid spacing of 0.16 nm. Coulomb and van der Waals potentials had cut off ratios of 1.2 nm. Production runs were performed in triplicate with a 2 fs time-step. For COX-1, simulations were carried out for 200 ns, while for COX-2 production runs were carried out for 250 ns.

### 3.4. Analysis of MD Simulation Trajectories

Analysis of trajectories was performed using tools included within the GROMACS 2018.2 software package [48,49]. *gmx rms* was used to calculate the root mean square deviation (RMSD) of the protein backbone and ligand, *gmx rmsf* was used to calculate the root mean square fluctuation (RMSF), *gmx gyrate* was used for the radius of gyration (Rg), *gmx sasa* was used to calculate the solvent accessible surface area, and *gmx hbond* was used to calculate the number of hydrogen bonds and number of pairs (cut-off = 0.35 nm) between the ligand and protein. Complexes were visualized using VMD 1.9.3 [70].

To calculate the binding free energy of olive-derived compounds to COX-1 and COX-2, molecular mechanics Poisson–Boltzmann surface area (MM-PBSA) was calculated using g_mmpbsa [79]. Calculations were performed on the final 20 ns of the trajectories at 80 ps intervals in triplicate on each monomer of the COX isoenzymes separately. The adaptive Poisson–Boltzmann solver (APBS) was used to calculate energy contributions from electrostatic, van der Waals, and polar solvation terms [80]. Grid spacing was set to 0.05 nm, with values of 80 and 2 used for the solvent and solute dielectric constants. The non-polar energy contribution was estimated by solvent-accessible surface area (SASA) with a probe radius of 0.14 nm. Entropic energy terms were excluded from calculations.

### 3.5. In Vitro Inhibitory Activity

The inhibitory activity of a selection of compounds against COX-1 (Abcam, BioVision ab204698, Waltham, MA, USA), COX-2 (Abcam, BioVision ab283401, USA), and 15-LOX (Cayman Chemical, 760700, Ann Arbor, MI, USA) was measured using commercially available inhibitor screening kits according to the manufacturer’s instructions. Briefly, 10 µL of each test inhibitor was added to 80 µL of COX reaction mix in each well. To initiate the reaction, 10 µL of diluted arachidonic acid solution was added to each well. Fluorescence was read on a CLARIOstar Microplate Reader (BMG Labtech, Ortenberg, Germany) at Ex/Em = 535/587 nm in kinetic mode for 10 min at 25 °C. For the 15-LOX assay, 10 µL of each test inhibitor was added to 90 µL of 15-LOX in each well. To initiate the reaction, 10 µL of arachidonic acid solution was added to each well. After incubation, chromogen (100 µL) was rapidly added to stop enzyme catalysis. The absorbance was read at 490–500 nm using the CLARIOstar Microplate Reader (BMG Labtech, Ortenberg, Germany).

The relative inhibition (%) at a concentration of 12.5 µM was recorded. The compounds OLC, HT, OLE, TYR, HVA, OA, LA, and PA were sourced from Sigma-Aldrich (St. Louis, MO, USA). HTA was obtained from Enzo Life Sciences (Farmingdale, NY, USA). OLP was synthesized by Occhem Labs, LLC (Oakdale, MN, USA), as previously described [29]. The data presented denotes the mean ± standard deviation (SD) from duplicate assays. A two-way analysis of variance (ANOVA) was conducted, followed by Šídák’s multiple comparison test to determine statistical significance for COX-1 and COX-2.

The potential inhibitory activity of verapamil, OLC, and OLP was tested using human ether-à-go-go-related gene (hERG) patch clamp assays (QPatch; performed by Reaction Biology Corp, Malvern, PA, USA). Verapamil was used as the positive control. The compounds were tested in 6-dose IC_50_ mode with 3-fold serial dilution in triplicate starting at 100 µM for OLP and 10 µM for OLC and verapamil (Figure S5). The data presented denote the mean ± SD (representative results from two independent experiments). A two-way analysis of variance (ANOVA) was conducted, followed by Dunnett’s multiple comparison test to determine statistical significance. The graphs were produced using GraphPad Prism 9.5.1 (GraphPad Software, San Diego, CA, USA).

### 3.6. In Silico Prediction of Pharmacokinetic Properties

The ADMET profile of OLC and OLP were predicted using SwissADME and QikProp (Schrödinger Suite) [53,54]. The properties of interest included gastrointestinal (GI) absorption, human oral absorption, Caco-2 cell permeability, BBB permeability, P-gp substrate prediction, inhibition of the CYP450 enzymes, and hERG inhibition. The reference values and results from the QikProp analysis can be found in Table S2.

### 3.7. Steered MD and Umbrella Sampling to Determine Membrane Permeability

Steered MD simulations (SMD) were performed to assess the membrane permeability of olive-derived compounds as previously described [56]. Briefly, SMD was performed using the GROMACS 4.6.5 software package [49,50] using the CHARMM27 force field [49,50]. The 1,2-dioleyl-sn-glycero-3-phosphocholine (DOPC) membrane topology was obtained from Lipidbook [81], consisting of 72 lipids per leaflet and spanning the *x*–*y* membrane in a continuous manner. The system was hydrated with the TIP3P water model [74] and the ligand was positioned ~1.5 nm above the membrane. Pulling simulations were performed under the NPT ensemble with a pull rate of 0.01 nm/ps for 800 ps. Ten independent runs were performed for each ligand.

Umbrella sampling was performed to calculate the potential of mean force (PMF) profile for OLP. OLP was constrained at 32 different locations along the bilayer normal (z) spaced approximately 0.2 nm apart and allowed to move unconstrained in the *x*–*y* plane. Each position along the bilayer normal was explored in a separate simulation with a run time of 100 ns for each window. The PMF profile was calculated using the Weighted Histogram Analysis Method (WHAM) [82], in GROMACS as *g_wham* [83].

## 4. Conclusions

Overall, the mechanisms underlying the anti-inflammatory properties of bioactive olive-derived compounds were investigated. The enzymatic assays demonstrated that phenolic compounds and fatty acids inhibit the activity of COX-1 and COX-2 enzymes, with minimal inhibitory activity against 15-LOX. Molecular docking results revealed that OLC and OLP bind to the active sites of the COX enzymes, forming stable interactions with key residues within inhibitory domains as highlighted MD simulations. Favorable pharmacokinetic properties were observed, and patch clamp assays revealed OLC and OLP to be non-inhibitors of the hERG channel. Our results extend knowledge of anti-inflammatory mechanisms associated with compounds derived from *Olea europaea*. In this context, further evaluation of novel olive-derived compounds and perhaps combinations as potential anti-inflammatories is warranted.

## Figures and Tables

**Table 1:** Predicted hydrogen bonds (H-bond) and π–π stacking interactions between olive-derived compounds and the active site of COX enzymes.

		COX-1	COX-2
OLC	Chain A	F209: π–π stacking	R120: H-bond Y385: H-bond
	Chain B	F209: π–π stacking Y385: H-bond	Y355: H-bond
OLP	Chain A	R120: H-bond	R120: H-bond Y355: H-bond
	Chain B	R120: H-bonds Y355: H-bond	R120: H-bonds G526: H-bond
LA	Chain A	V349: H-bond	R120: H-bond
	Chain B	-	R120: H-bond

**Table 2:** Binding free energy contribution terms for OLC and OLP bound to two chains of COX-1 and COX-2. Energy terms are shown in kcal/mol and calculated as mean ± SD of three independent simulations.

Ligand	ΔE_vdW_	ΔE_elec_	ΔG_polar_	ΔG_nonpolar_	ΔG_bind_
COX-1					
OLC-A	−40.36 ± 2.84	−8.04 ± 1.73	29.38 ± 2.89	−4.55 ± 0.09	−23.57 ± 1.99
OLC-B	−42.80 ± 1.08	−7.06 ± 0.92	32.62 ± 2.27	−4.60 ± 0.08	−21.83 ± 0.90
OLP-A	−38.44 ± 0.87	−10.96 ± 1.82	35.95 ± 3.21	−4.10 ± 0.10	−17.55 ± 0.56
OLP-B	−37.30 ± 0.53	−13.46 ± 5.90	39.15 ± 4.31	−4.21 ± 0.04	−15.83 ± 1.75
COX-2					
OLC-A	−43.31 ± 0.55	−6.15 ± 1.27	31.63 ± 0.87	−4.67 ± 0.04	−22.51 ± 1.84
OLC-B	−39.08 ± 1.91	−6.76 ± 3.05	37.28 ± 4.20	−4.85 ± 0.18	−13.45 ± 1.45
OLP-A	−37.42 ± 3.46	−13.50 ± 6.49	38.95 ± 7.47	−4.20 ± 0.12	−16.15 ± 1.52
OLP-B	−40.22 ± 0.83	−13.08 ± 0.87	39.11 ± 3.15	−4.08 ± 0.06	−18.28 ± 1.59

Abbreviations: ΔE_vdW_ = van der Waals interaction, ΔE_elec_ = electrostatic interaction, ΔG_polar_ = polar contribution, ΔG_nonpolar_ = non-polar contribution to the solvation free energy estimated by solvent-accessible surface area (SASA), ΔG_binding_ = binding free energy.

## Data Availability

The original contributions presented in the study are included in the article/Supplementary Materials, further inquiries can be directed to the corresponding author.

## Supplementary Materials

The following supporting information can be downloaded at: https://www.mdpi.com/article/10.3390/molecules29153502/s1, Figure S1. Interactions of olive-derived compounds with the active site of COX-1. The predicted protein-ligand interactions of OLC, OLP, and LA are shown for the (A–C) chain A and (D–F) chain B subunits of homodimeric COX-1. Hydrophobic residues are colored green, positively charged residues are colored purple, polar residues are colored blue, and negatively charged residues are colored red. Hydrogen bonds and π–π stacking interactions are colored purple and green, respectively; Figure S2. Interactions of olive-derived compounds with the active site of COX-2. The predicted protein-ligand interactions of OLC, OLP, and LA are shown for the (A–C) chain A and (D–F) chain B subunits of homodimeric COX-2. Hydrophobic residues are colored green, positively charged residues are colored purple, polar residues are colored blue, and negatively charged residues are colored red. Hydrogen bonds and π–π stacking interactions are colored purple and green, respectively; Figure S3. Interactions of olive-derived compounds with the active site of 15-LOX. The predicted interactions of (A) OLC, (B) OLP, and (C) LA with 15-LOX are shown. Hydrophobic residues are colored green, positively charged residues are colored purple, polar residues are colored blue, and negatively charged residues are colored red. Hydrogen bonds and π–π stacking interactions are colored purple and green, respectively; Figure S4. Heatmap of all residues contributing to binding energy for OLC and OLP bound to each chain of (A) COX-1 and (B) COX-2 homodimers. Energy contributions are shown in kcal/mol as an average of three independent binding free energy calculations using MM-PBSA; Figure S5. Inhibition of the human ether-à-go-go related gene (hERG) K+ channels by olive-derived phenolic compounds. The results are shown for 0.37, 1.11, 3.33, and 10.00 µM. Verapamil was utilized as a positive control and inhibited 100% of hERG activity at a concentration of 10 µM. The phenolic compounds OLC and OLP were found to inhibit 44% and 10% of hERG activity at concentrations of 10 and 100 µM, respectively. The data presented denote the mean ± SD from triplicate assays (representative results from two independent experiments). ** *p* ≤ 0.01, *** *p* ≤ 0.001, and **** *p* ≤ 0.0001 quantified using a 2-way ANOVA with Dunnett’s multiple comparisons test; Figure S6. Unsymmetrized PMF curves for OLP passing through a DOPC membrane along the bilayer normal with progressive increments of production runs discarded for equilibration; Figure S7. Homology model of the human arachidonate 15-lipoxygenase (15-LOX). (A) The Ramachandran plot is provided for the open conformation of the human 15-LOX structure. PROCHECK was used to assess the stereochemical quality of the model. For the open conformation, 93.2% of residues were found to be in the most favored regions, 6.7% were in the allowed regions, and 0.2% were in the disallowed regions. (B) The N-terminal β-barrel and C-terminal α-helical catalytic domains are labelled; Table S1. Average relative inhibition (%) ± standard deviation (SD) of cyclooxygenase isoforms (COX-1 and COX-2) by *Olea europaea* compounds; Table S2. Pharmacokinetic properties of the phenolic compounds OLC and OLP predicted by the in silico tools QikProp and SwissADME.
