# Supplementary material for: Investigation of the Anti-Inflammatory Properties of Bioactive Compounds from Olea europaea: In Silico Evaluation of Cyclooxygenase Enzyme Inhibition and Pharmacokinetic Profiling
Source: Molecules. 2024 Jul 26;29(15):3502. doi: 10.3390/molecules29153502 (PMC11314539; doi:10.3390/molecules29153502)
Supplement: Supplementary file 1 [file molecules-29-03502-s001.zip › molecules-3077710-supplementary.pdf]

Investigation of the Anti-Inflammatory Properties of Bioactive Compounds from *Olea europaea*: In Silico Evaluation of Cyclooxygenase Enzyme Inhibition and Pharmacokinetic Profiling

Tom C. Karagiannis [1,2,3,4,*], Katherine Ververis [2,3], Julia J. Liang [1,2,5], Eleni Pitsillou [2,5], Evan A. Kagarakis [2], Debbie T. Z. Yi [2], Vivian Xu [2], Andrew Hung [5] and Assam El-Osta [1,6,7,8,9,10]

1 Epigenetics in Human Health and Disease Program, Baker Heart and Diabetes Institute, 75 Commercial Road, Prahran, VIC 3004, Australia
2 Epigenomic Medicine Laboratory at prospED Polytechnic, Carlton, VIC 3053, Australia
3 Department of Clinical Pathology, The University of Melbourne, Parkville, VIC 3010, Australia
4 Department of Microbiology and Immunology, The University of Melbourne, Parkville, VIC 3010, Australia
5 School of Science, STEM College, RMIT University, Melbourne, VIC 3001, Australia
6 Department of Diabetes, Central Clinical School, Monash University, Melbourne, VIC 3004, Australia
7 Department of Medicine and Therapeutics, The Chinese University of Hong Kong, Sha Tin, Hong Kong SAR, China
8 Hong Kong Institute of Diabetes and Obesity, Prince of Wales Hospital, The Chinese University of Hong Kong, 3/F Lui Che Woo Clinical Sciences Building, 30–32 Ngan Shing Street, Sha Tin, Hong Kong SAR, China
9 Li Ka Shing Institute of Health Sciences, The Chinese University of Hong Kong, Sha Tin, Hong Kong SAR, China
10 Biomedical Laboratory Science, Department of Technology, Faculty of Health, University College Copenhagen, 2200 Copenhagen, Denmark
* Correspondence: karat@unimelb.edu.au; Tel.: +61-3-8532-1290; Fax: +61-3-8532-1100

Table of Contents

Figure S1. Interactions of olive-derived compounds with the active site of COX-1…….... 2

Figure S2. Interactions of olive-derived compounds with the active site of COX-2…….... 3

Figure S3. Interactions of olive-derived compounds with the active site of 15-LOX……. 4

Figure S4. Heatmap of all residues contributing to binding energy for OLC and OLP bound to each chain of A) COX-1 and B) COX-2 homodimers…………………………………….. 5

Figure S5. Inhibition of the human ether-à-go-go related gene (hERG) K^+ channels by olive-derived phenolic compounds……………………………………………………………. 6

Figure S6. Unsymmetrized PMF curves for OLP passing through a DOPC membrane along the bilayer normal with progressive increments of production runs discarded for equilibration………………………………………………………………………….... 7

Figure S7. Homology model of the human arachidonate 15-lipoxygenase (15-LOX)…… 8

Table S1. Average relative inhibition (%) ± standard deviation (SD) of cyclooxygenase isoforms (COX-1 and COX-2) by *Olea europaea* compounds…………………………… 9

Table S2. Pharmacokinetic properties of the phenolic compounds OLC and OLP predicted by the *in silico* tools QikProp and SwissADME…………………………………………... 10

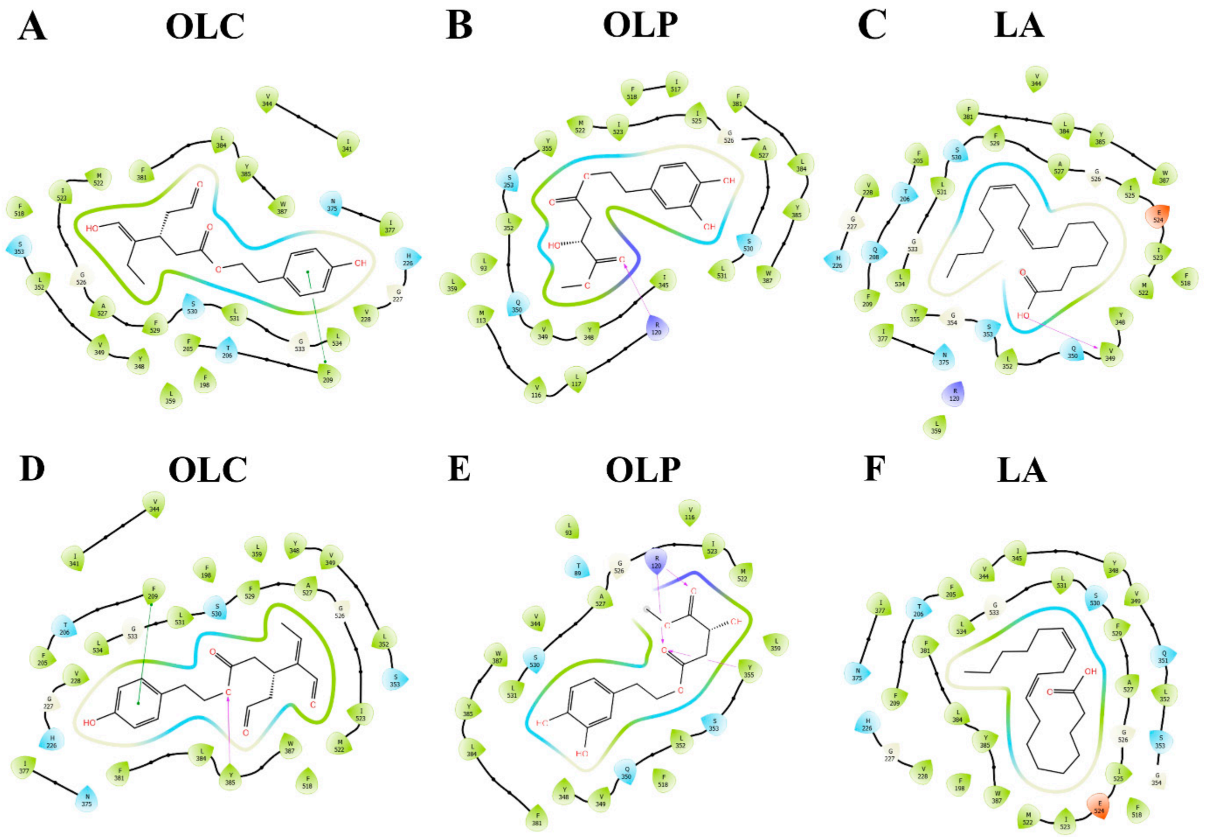

Figure S1. Interactions of olive-derived compounds with the active site of COX-1. The predicted protein-ligand interactions of OLC, OLP, and LA are shown for the (A-C) chain A and (D-F) chain B subunits of homodimeric COX-1. Hydrophobic residues are colored green, positively charged residues are colored purple, polar residues are colored blue, and negatively charged residues are colored red. Hydrogen bonds and π-π stacking interactions are colored purple and green, respectively.

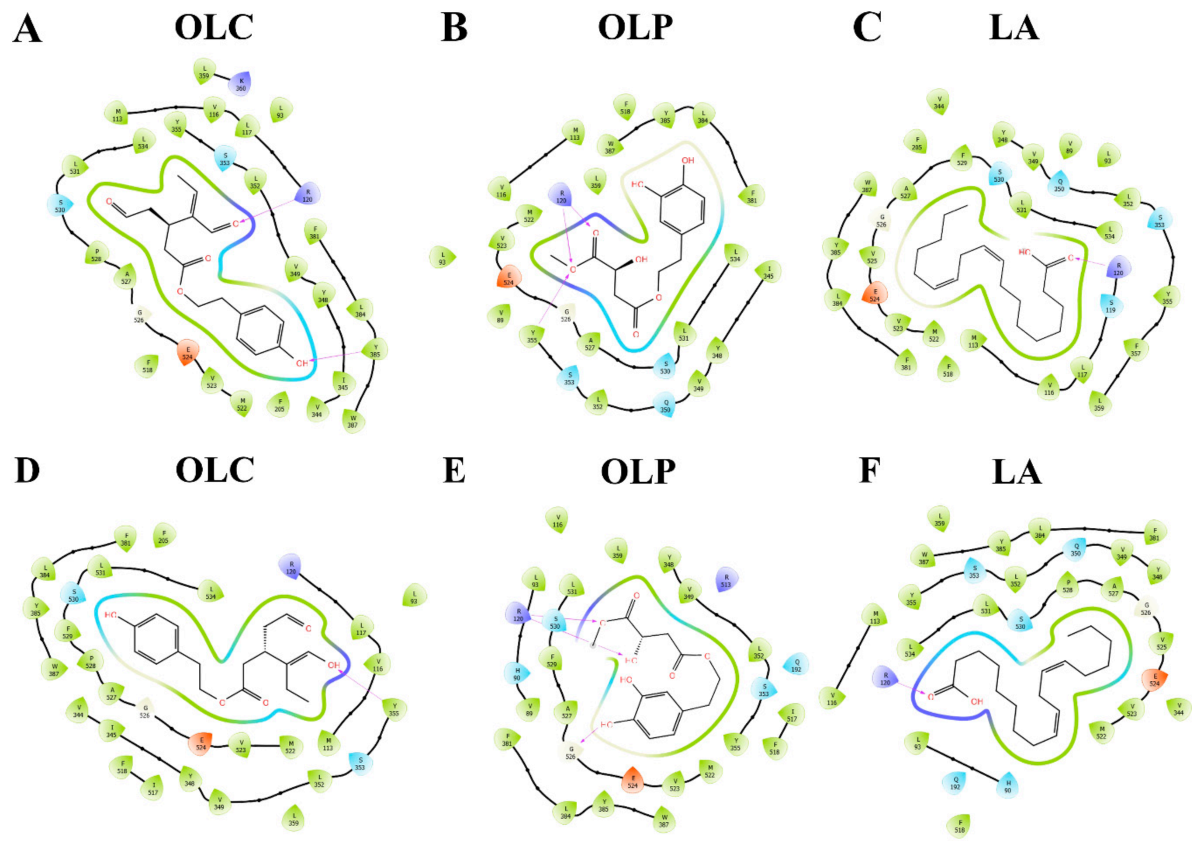

Figure S2. Interactions of olive-derived compounds with the active site of COX-2. The predicted protein-ligand interactions of OLC, OLP, and LA are shown for the (A-C) chain A and (D-F) chain B subunits of homodimeric COX-2. Hydrophobic residues are colored green, positively charged residues are colored purple, polar residues are colored blue, and negatively charged residues are colored red. Hydrogen bonds and π-π stacking interactions are colored purple and green, respectively.

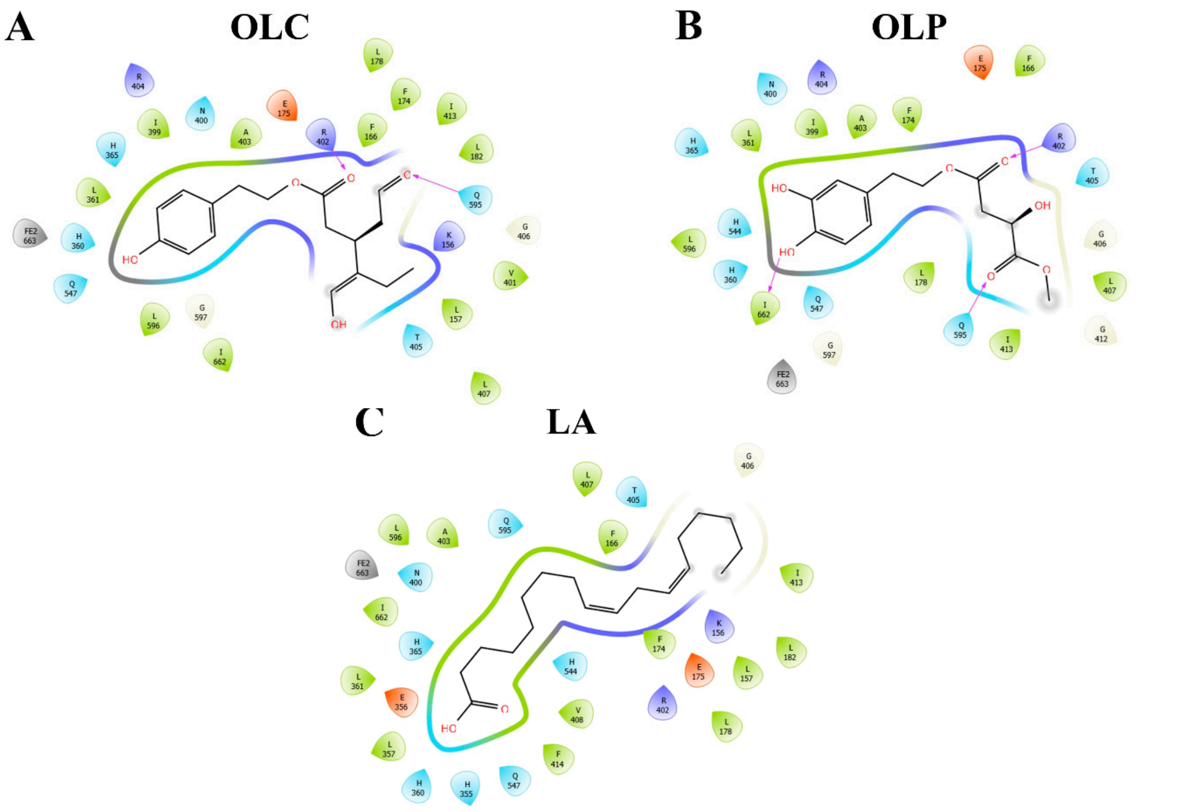

Figure S3. Interactions of olive-derived compounds with the active site of 15-LOX. The predicted interactions of (A) OLC, (B) OLP, and (C) LA with 15-LOX are shown. Hydrophobic residues are colored green, positively charged residues are colored purple, polar residues are colored blue, and negatively charged residues are colored red. Hydrogen bonds and π-π stacking interactions are colored purple and green, respectively.

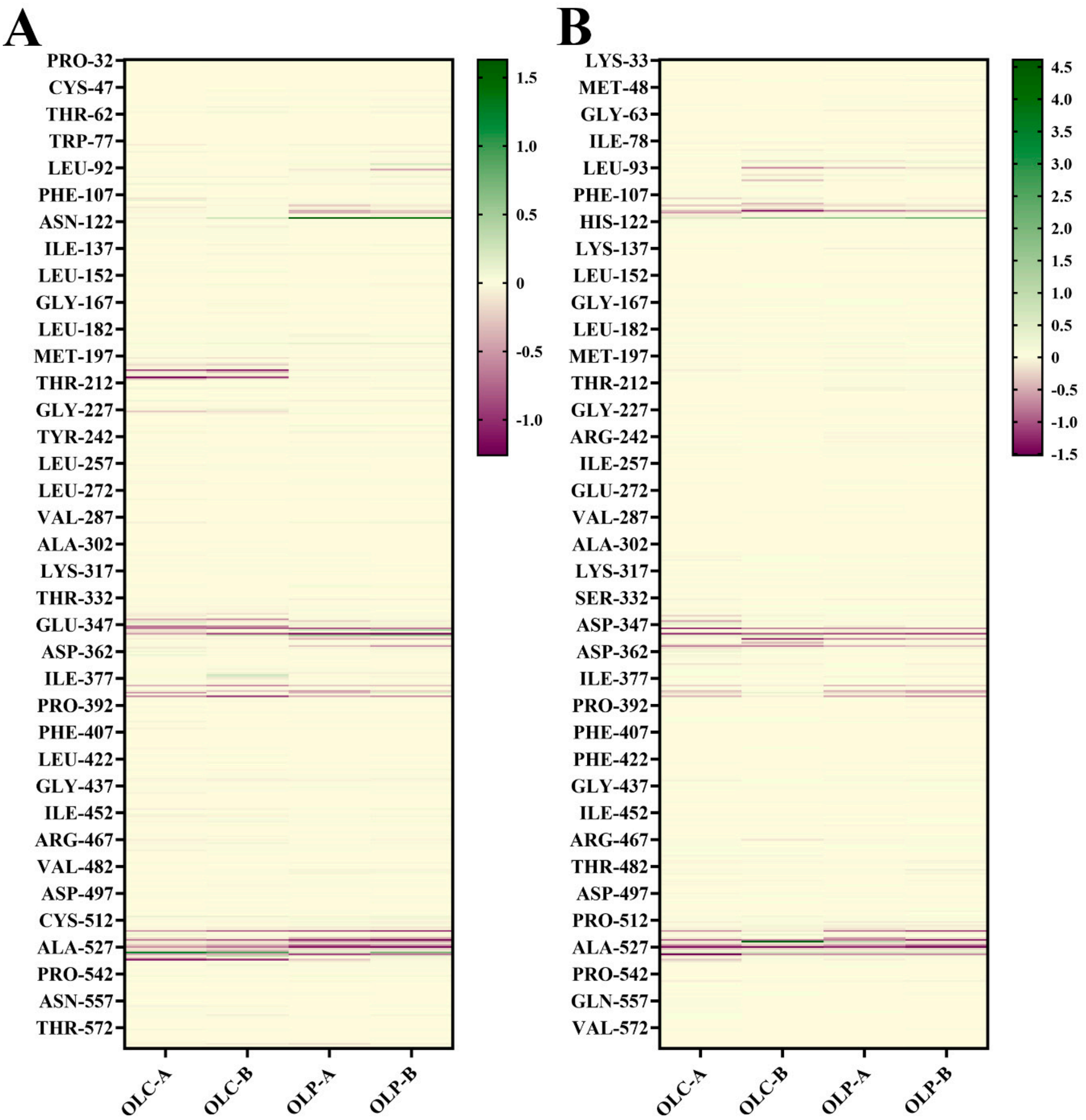

Figure S4. Heatmap of all residues contributing to binding energy for OLC and OLP bound to each chain of A) COX-1 and B) COX-2 homodimers. Energy contributions are shown in kcal/mol as an average of three independent binding free energy calculations using MM-PBSA.

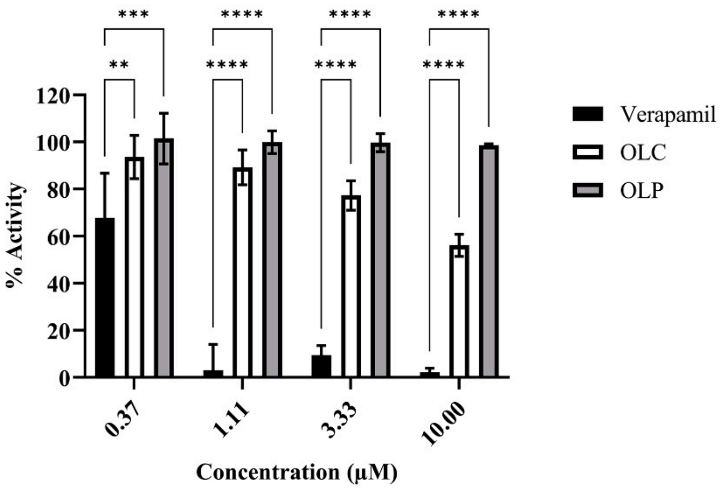

Figure S5. Inhibition of the human ether-à-go-go related gene (hERG) K^+ channels by olive-derived phenolic compounds. The results are shown for 0.37, 1.11, 3.33, and 10.00 µM. Verapamil was utilized as a positive control and inhibited 100% of hERG activity at a concentration of 10 µM. The phenolic compounds OLC and OLP were found to inhibit 44% and 10% of hERG activity at concentrations of 10 and 100 µM, respectively. The data presented denote the mean ± SD from triplicate assays (representative results from two independent experiments). ** $p \leq 0.01$, *** $p \leq 0.001$, and **** $p \leq 0.0001$ quantified using a 2-way ANOVA with Dunnett's multiple comparisons test.

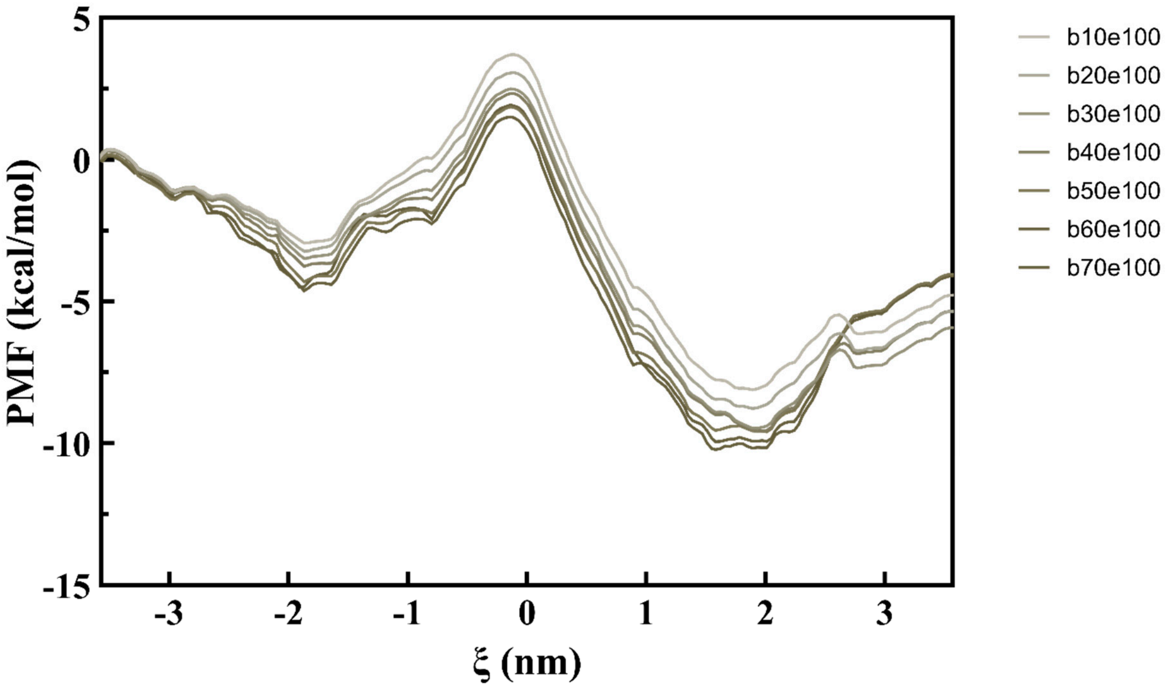

Figure S6. Unsymmetrized PMF curves for OLP passing through a DOPC membrane along the bilayer normal with progressive increments of production runs discarded for equilibration.

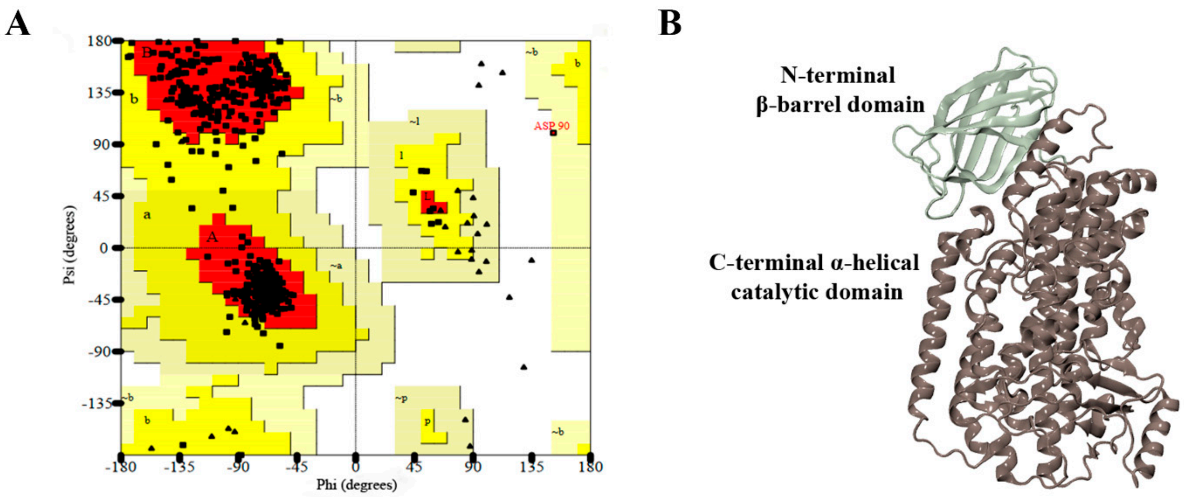

Figure S7. Homology model of the human arachidonate 15-lipoxygenase (15-LOX). (A) The Ramachandran plot is provided for the open conformation of the human 15-LOX structure. PROCHECK was used to assess the stereochemical quality of the model. For the open conformation, 93.2% of residues were found to be in the most favored regions, 6.7% were in the allowed regions, and 0.2% were in the disallowed regions. (B) The N-terminal β-barrel and C-terminal α-helical catalytic domains are labelled.

Table S1. Average relative inhibition (%) ± standard deviation (SD) of cyclooxygenase isoforms (COX-1 and COX-2) by *Olea europaea* compounds.

Compound	COX-1 Inhibition (%)[a]	COX-2 Inhibition (%)[b]	15-LOX Inhibition (%)[c]
	12.5µM	12.5µM	12.5 µM
**OLP**	24.4 ± 5.5	18.8 ± 1.4	3.3 ± 4.6
**OLC**	26.6 ± 1.7	21.8 ± 0.4	0.2 ± 7.6
**HT**	25.5 ± 6.7	29.4 ± 2.5	0.0 ± 0.0
**HTA**	34.8 ± 7.3	19.6 ± 1.9	0.0 ± 0.0
**OLE**	28.9 ± 5.1	30.1 ± 2.7	5.3 ± 3.1
**TYR**	26.2 ± 1.3	21.1 ± 2.7	0.0 ± 0.0
**HVA**	0.0 ± 0.0	14.4 ± 3.0	15.7 ± 6.2
**OA**	25.3 ± 5.0	7.0 ± 5.8	2.8 ± 8.6
**PA**	22.5 ± 4.2	17.2 ± 0.5	2.6 ± 3.6
**LA**	67.0 ± 0.2	81.4 ± 0.7	0.0 ± 0.0

[a] Positive control in the COX-1 assay SC560 (1nM) inhibited 75 ± 3.1 %

[b] Positive control in the COX-2 assay celecoxib (1µM) inhibited 91 ± 0.4 %

[c] Positive control in the 15-LOX assay NDGA (100µM) inhibited 100%

Table S2. Pharmacokinetic properties of the phenolic compounds OLC and OLP predicted by the *in silico* tools QikProp and SwissADME.

		**OLC**	**OLP**
**QikProp**	QPPCaco:<br>• Predicted apparent Caco-2 cell permeability in nm/sec (model for gut-blood barrier)<br>• <25 poor, >500 great	107.6	38.3
	QPlogBB:<br>• Predicted brain/blood partition coefficient (for orally delivered drugs)<br>• −3.0 - 1.2	-2.1	-2.4
	QPPMDCK<br>• Predicted apparent MDCK cell permeability in nm/sec (model for the blood-brain barrier)<br>• <25 poor, >500 great	44.4	14.5
	Percent human oral absorption<br>• >80% is high, <25% is poor	74.7	60.3
	QPloghERG<br>• Predicted IC_{50} value for blockage of hERG K^+ channels<br>• Concern below -5	-5.2	-4.7
**SwissADME**	GI absorption	High	High
	BBB permeation	No	No
	P-gp substrate	No	No
	CYP1A2 inhibitor	No	No
	CYP2C19 inhibitor	No	No
	CYP2C9 inhibitor	No	No
	CYP2D6 inhibitor	No	No
	CYP3A4 inhibitor	No	No
